# Association between gestational weight gain and adverse pregnancy outcomes: a systematic review and meta-analysis

**DOI:** 10.3389/fgwh.2026.1777069

**Published:** 2026-04-28

**Authors:** Xiaohong Ye, Peishan Li, Chuan Shi

**Affiliations:** Obstetrics, Chengdu Shuangliu District Maternal and Child Health Hospital, Chengdu City, Sichuan Province, China

**Keywords:** cesarean section, gestational weight gain, meta-analysis, preeclampsia, pregnancy outcomes

## Abstract

**Background:**

Abnormal gestational weight gain (GWG) has been associated with a wide range of adverse maternal and neonatal outcomes; however, existing evidence remains inconsistent, particularly across different regions and levels of national development. Moreover, the applicability of commonly used GWG guidelines in diverse populations, especially in developing countries, remains unclear. This systematic review and meta-analysis aimed to comprehensively evaluate the association between abnormal GWG and adverse pregnancy outcomes using multinational cohort data.

**Methods:**

We conducted a systematic review and meta-analysis of cohort studies published from 2009 to April 2025. Studies defining GWG by IOM/WHO/ACOG guidelines and reporting outcomes like cesarean section, preterm birth, preeclampsia, macrosomia, or LGA/SGA were included. Data extraction and quality assessment (via NOS) were performed independently by reviewers. Pooled ORs with 95% CIs were calculated using fixed/random-effects models in Stata.

**Results:**

Seventeen studies involving 866,593 pregnant women were included. Compared with adequate GWG, excessive GWG was associated with significantly increased risks of cesarean section (OR = 1.32, 95% CI: 1.23–1.42), preeclampsia (OR = 2.05, 95% CI: 1.67–2.52), macrosomia (OR = 2.18, 95% CI: 2.03–2.35), and LGA (OR = 2.20, 95% CI: 1.97–2.46), while no significant association was observed with preterm birth or SGA. In contrast, inadequate GWG was associated with an increased risk of preterm birth (OR = 1.45, 95% CI: 1.08–1.94) but lower risks of cesarean section (OR = 0.89, 95% CI: 0.82–0.98), macrosomia (OR = 0.70, 95% CI: 0.55–0.91), and LGA (OR = 0.65, 95% CI: 0.56–0.75). Substantial regional and developmental differences were observed, with stronger associations generally found in developing countries.

**Conclusion:**

Our findings highlight that the effects of abnormal GWG are highly context-dependent, varying substantially by region, national development level, and guideline standards. These results underscore the need for population-specific GWG recommendations and targeted gestational weight management strategies, particularly in developing countries.

## 1 Introduction

Gestational weight gain (GWG) is a crucial indicator for assessing nutritional status during pregnancy. It is defined as the difference between a woman's pre-pregnancy weight and her weight measured at delivery (1). Based on a substantial body of evidence, the Institute of Medicine (IOM) revised the GWG guidelines in 2009, clearly categorizing the recommended range according to pre-pregnancy body mass index (Body Mass Index, BMI) as follows: underweight (BMI < 18.5 kg/m^2^) 12.5–18.0 kg, normal weight (18.5–24.9 kg/m^2^) 11.5–16.0 kg, overweight (25.0–29.9 kg/m^2^) 7.0–11.5 kg, and obesity (≥30.0 kg/m^2^) 5.0–9.0 kg (1). However, global statistics indicate a widespread issue of abnormal GWG. For example, a study in the United States reported that the incidence of excessive GWG ranged from 38.2%–54.7% (2), with similar data in China ranging from 37% to 50.9% (3, 4). Similarly, insufficient GWG occurs in approximately 20.9% of cases in the U.S (2)., and in China, it is reported to be between 15.6% and 32.8% (3, 4).

Abnormal GWG is closely associated with various adverse maternal and infant outcomes. GWG arises from fetal development, maternal uterine enlargement, breast development, increased blood volume, body fluid, and fat storage (5). Achieving a satisfactory GWG is vital for the successful completion of pregnancy (6). Excessive GWG significantly increases the risk of large-for-gestational-age (LGA) infants, macrosomia, preeclampsia, gestational diabetes, and cesarean section (C-section) (7–9). On the other hand, insufficient GWG is significantly associated with low birth weight (10, 11) and preterm labor (12). Notably, the long-term effects of abnormal GWG may persist into adulthood for the offspring, including an increased risk of obesity, elevated triglyceride levels (13), and long-term BMI after birth (14, 15).

Despite existing evidence indicating that GWG is associated with pregnancy outcomes, key controversies and gaps remain to be clarified. First, the conclusions of multiple systematic reviews are inconsistent, for instance, the study by Choi SK et al. (16) found that pre-pregnancy overweight and obesity were more strongly associated with adverse obstetric outcomes than weight gain during pregnancy. Moreover, insufficient weight gain during pregnancy can lead to severe complications, while the research by Santos S et al. (9) showed that maternal pre-pregnancy BMI and GWG were associated with the risk of pregnancy complications across the entire range. Obese mothers with high GWG had the highest risk of pregnancy complications. Furthermore, existing evidence has notable limitations: on one hand, most studies have not adequately considered the variability in the applicability of the International Medical Organization (IOM) standards across different populations; on the other hand, systematic reviews generally lack sample data from populations in developing countries (17), resulting in insufficient generalizability of conclusions on a global scale. In summary, current research has yet to elucidate the independent role of GWG abnormalities and their interaction with pre-pregnancy BMI under a unified standard based on diverse global data, constituting a critical evidence gap in this field that urgently needs to be addressed (18).

Therefore, this study aims to conduct a systematic review and meta-analysis to assess and clarify the relationship between abnormal GWG and adverse pregnancy outcomes. We innovatively integrated international cohort data from multiple countries, including developing nations, for subgroup analysis for the first time. The study specifically explores the applicability differences of various GWG guideline standards across diverse populations, the impact of abnormal GWG on pregnancy outcomes, and its relationship with national development levels. The anticipated outcomes will not only provide more precise and context-specific evidence to support GWG management but also promote greater scientific emphasis on gestational weight management in clinical and public health practices, ultimately offering evidence-based insights to improve global maternal and infant health outcomes.

## 2 Methods

This study was conducted in accordance with the Preferred Reporting Items for Systematic Reviews and Meta-Analyses (PRISMA) guidelines and was registered in the PROSPERO database (ID: CRD 42025101867). The data for this study were derived from previously published articles and did not involve the collection of new patient information, therefore, ethical approval or patient consent was not required.

### 2.1 Search strategy

Two researchers (YXH and SC) independently searched the databases PubMed, Cochrane, Embase, and Web of Science. We used combinations of free text and medical subject heading terms: “Gestational Weight Gain”, “Pregnancy Outcome”, “Pregnancy Complications”, “Maternal Weight Gain”, “Pregnancy Weight Gain”, “Postpartum Weight Retention”, “Pregnancy Outcome*”, “birth outcome”, “obstetric outcome”, “Pregnancy Complication”, “Adverse Birth Outcome*”, “complications related to pregnancy”, “gestational complication*”, “pregnancy neoplastic complications” and “pregnancy-associated complications” These terms were connected using Boolean logic operators to construct the search query. The search strategy was adjusted according to the specific requirements of each database, and the complete search strategy can be found in the supplementary materials. The search period was from the inception of each database to April 8, 2025. All citations were directly retrieved from the respective databases. In addition, we manually searched clinical trial registry platforms and the reference lists of related articles to avoid missing relevant studies.

### 2.2 Inclusion and exclusion criteria

Inclusion Criteria include: (1) Published in English; (2) Since the IOM guidelines were revised in 2009, we selected literature published after 2009; (3) Single or multiple pregnancies (twins); (4) Reported complete data on at least one outcome of interest to us; (5) Definition of GWG categories: Gestational weight gain was classified as “inadequate,” “adequate,” or “excessive” according to validated international guidelines. For studies following the IOM (2009) and American College of Obstetricians and Gynecologists (ACOG) (2013) criteria, adequacy was defined based on pre-pregnancy BMI: 12.5–18.0 kg for underweight (<18.5 kg/m^2^), 11.5–16.0 kg for normal weight (18.5–24.9 kg/m^2^7.0–11.5 kg for overweight (25.0–29.9 kg/m^2^), and 5.0–9.0 kg for obese (≥30.0 kg/m^2^). Studies employing World Health Organization (WHO) standards followed similar BMI-based weight ranges. Total weight gain falling below or exceeding these specific ranges was defined as inadequate or excessive GWG, respectively (6); Pre-pregnancy BMI categorized according to WHO classification as underweight, normal weight, overweight, or obese.

Exclusion Criteria include: (1) Studies without abstracts; (2) Studies that do not report relevant data on key outcomes; (3) Systematic reviews, meta-analyses, case reports, conference abstracts, animal experiments, or letters; (4) Studies with unavailable full-text articles; (5) Studies focusing on twin or multiple pregnancies were excluded to avoid bias, as weight gain patterns and clinical recommendations for multiple gestations differ significantly from those for singleton pregnancies. Consequently, all participants included in this meta-analysis were individuals with singleton pregnancies.

### 2.3 Literature screening and data extraction

Two researchers (YXH and SC) managed all the literature using Endnote X9 software. After importing the retrieved articles, duplicates were removed, and studies that met the inclusion and exclusion criteria were screened based on the title and abstract. Full-text articles were then downloaded, and after reviewing the full text, studies that met the requirements for this systematic review and meta-analysis were selected.

The basic information of the literature was extracted and cross-checked, with a unified measurement unit. If there were any disagreements, a third researcher (LPS) was consulted to discuss and resolve the differences. The extracted information mainly included the title, first author, publication year, study design, country of authors, country of research subjects, sample size, patient age, GWG reference standard, major disease background, and outcome indicators.

### 2.4 Literature quality assessment

Two researchers (YXH and SC) independently evaluated the quality of the literature based on the inclusion and exclusion criteria for initial screening. For studies with discrepancies, a third researcher (LPS) made the final judgment to resolve conflicts, and the final decision was discussed and agreed upon. To assess methodological quality and bias risk, we used the Newcastle-Ottawa Scale (NOS), which is suitable for evaluating observational studies. The scale consists of three main components: selection of study participants, comparability, and outcome measurement. The NOS uses a star rating system (⭐) with a maximum score of nine stars. The higher the score, the higher the study quality. Scores of 1–3 represent low quality, 4–6 represent moderate quality, and 7–9 represent high quality.

### 2.5 Outcome indicators

Primary Outcome Indicators: ① Cesarean Section: Refers to a surgical procedure in which the abdominal wall and uterus are incised to deliver the fetus; ② Preterm Labor: Refers to delivery between 28 and 37 weeks of gestation. Babies born at this gestational age are referred to as preterm; ③ Preeclampsia: A syndrome that occurs after 20 weeks of pregnancy, characterized by hypertension (≥140/90 mmHg) and proteinuria (≥0.3 g/24 h or random urine protein positive), which may be accompanied by multi-organ dysfunction.

Secondary Outcome Indicators: ① Macrosomia: Refers to a newborn whose birth weight exceeds 4,000 g; ② LGA (Large for Gestational Age): Refers to a newborn whose birth weight is greater than the 90th percentile (P90) for the same gestational age; ③ SGA (Small for Gestational Age): Refers to a newborn whose birth weight is less than the 10th percentile (P10) for the same gestational age.

This study selected cesarean section, preterm birth, and preeclampsia as primary pregnancy outcome indicators, while including macrosomia, large for gestational age (LGA), and small for gestational age (SGA) as secondary indicators. This approach was adopted because these indicators collectively cover critical dimensions of maternal and infant health: the primary indicators directly reflect severe complications threatening maternal and infant safety (preeclampsia) and important delivery outcomes (cesarean section, preterm birth), demonstrating clear clinical urgency. The secondary indicators systematically assess the spectrum of fetal growth and development, ranging from overnutrition (macrosomia/LGA) to growth restriction (SGA), thereby comprehensively capturing the long-term effects of abnormal gestational weight on fetal development. Moreover, all indicators adhere to internationally standardized diagnostic criteria, making them suitable for cross-healthcare-system comparisons and data integration, particularly aligning with the needs of multinational cohort meta-analyses.

### 2.6 Statistical analysis

Statistical analysis was conducted using StataSE (version 15.1; Stata Corp, College Station, TX, USA). For binary variables related to outcomes (i.e., occurrence or non-occurrence), the event counts were entered, and odds ratios (OR) with corresponding 95% confidence intervals (CIs) were calculated. If both unadjusted OR and adjusted OR (AOR) were reported, adjusted ORs and their 95% CIs were prioritized. Heterogeneity between studies was assessed using the I² statistic. If *I*^2^ < 50%, indicating low heterogeneity, a fixed-effect model was applied for analysis; if *I*^2^ > 50%, indicating significant heterogeneity, a random-effect model was used.

To explore the potential sources of heterogeneity, subgroup analysis was performed based on the following variables: different countries/regions and different GWG reference standards. Funnel plots and Egger's test were used to assess publication bias. If the funnel plot showed asymmetry or if Egger's test yielded a *P*-value < 0.05, publication bias was indicated. Conversely, if the funnel plot was symmetrical and Egger's test yielded a *P*-value ≥ 0.05, no publication bias was detected, suggesting that the meta-analysis results were robust and reliable. Additionally, sensitivity analysis was performed using a leave-one-out method to examine the influence of any single study on the overall results. A *P*-value < 0.05 was considered statistically significant.

## 3 Results

### 3.1 Literature search process

**Figure 1:**
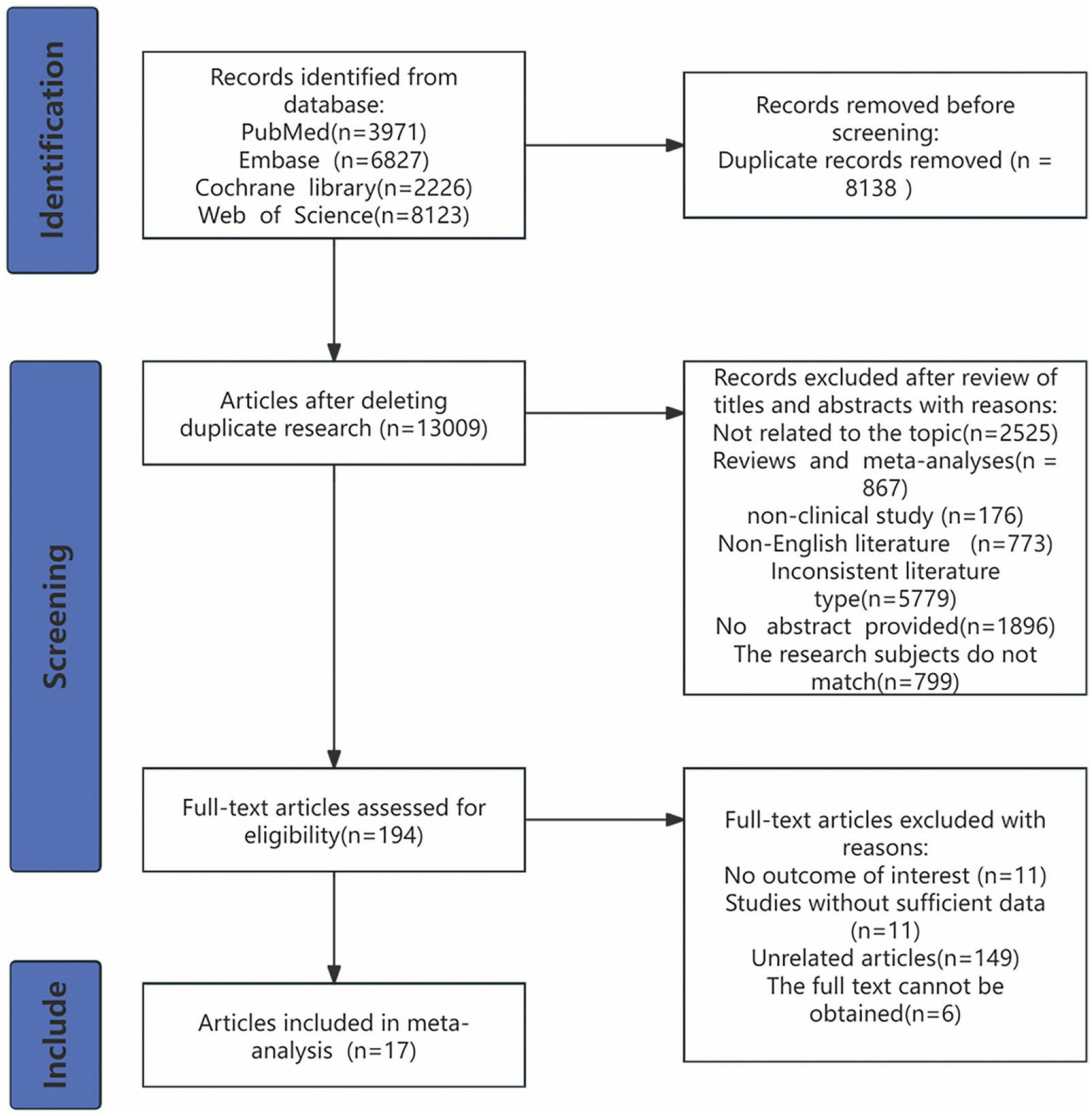
PRISMA flow diagram for article selection and screening.

A total of 21,147 articles were retrieved, including 3,971 from PubMed, 6,827 from Embase, 2,226 from The Cochrane Library, and 8,123 from Web of Science. After removing duplicate articles, 13,009 articles remained. These were screened to exclude studies unrelated to the research topic, systematic reviews, meta-analyses, case reports, conference papers, animal experiments, letters, non-clinical studies, non-English articles, articles with inconsistent document types, articles without abstracts, and studies with inappropriate research subjects. Following this, 194 articles underwent full-text assessment to determine whether they met the inclusion criteria. Further screening excluded studies with irrelevant outcomes, insufficient data, or unrelated content, leaving a total of 17 articles (17, 19–34) for inclusion. The detailed process and results of the literature search are shown in (Figure 1).

### 3.2 Basic characteristics of included studies

A total of 17 studies were included, with the research subjects categorized by geographical region as follows: 11 studies (23–31, 33, 34) included subjects from Asia (eight from China, two from Iran, one from Thailand, and one from Turkey), two studies (17, 19) involved subjects from Europe, and three studies (20, 22, 32) included subjects from North America (two from the United States and one from Mexico). Fifteen studies (17, 19, 21–33) used the IOM guidelines as the GWG reference standard, one study (20) used the WHO reference standard, and one study (34) followed the ACOG 2013 guidelines. The sample sizes ranged from 453 to 722,839 women, with a total of 866,593 women included across all 17 studies. The basic characteristics of the included studies are shown in (Table 1).

**Table 1:** The basic characteristics of the included studies.

Author	Year	Study design	Country	Sample size				Age			GWG reference standard	Outcomes
				Total	Above Group A	Normal Group B	Below Group C	Above Group A	Normal Group B	Below Group C		
Monteiro (19)	2022	retrospective study	Portuguese	8,040	2,170	2,492	3,378	32.2 ± 5.6	32.8 ± 5.3	33.3 ± 5.3	IOM guidelines	1. Preeclampsia 2. Cesarean section Preterm labor 3. SGA******* 4. LGA 5. Macrosomia
Rosinha (17)	2022	retrospective cohort study	Portuguese	453	149	147	157	NR	NR	NR	IOM guidelines	1. Preeclampsia 2. Cesarean delivery 3. LGA 4. Prematurity
Ukah, (20)	2019	retrospective cohort study	US	722,839	351,615	227,715	143,509	NR	NR	NR	WHO	C-section
Yazan Arslan (21)	2025	retrospective study	Türkiye	642	236	233	173	NR	NR	NR	IOM guidelines	1. Preterm 2. LGA 3. Macrosomia
Langford (22)	2011	cohort study	USA	34,143	25,151	7,205	1,787	NR	NR	NR	IOM guidelines	1. Preeclampsia 2. C-section 3. Macrosomia
Liu (23)	2015	retrospective study	China	2,973	1,600	969	404	NR	NR	NR	IOM guidelines	Cesarean section Preterm delivery Preeclampsia SGA Macrosomia
Gao (24)	2017	cohort study	China	919	384	364	171	29.7	30	30	IOM guidelines	1. LGA 2. *P*-CS (primary cesarean section)
Shi (25)	2014	retrospective study	China	15,986	6,291	6,278	3,417	NR	NR	NR	IOM guidelines	Macrosomia
Wang (26)	2018	prospective cohort study	China	601	222	267	112	NR	NR	NR	IOM guidelines	1. Macrosomia 2. LGA
Shao (27)	2017	cohort study	China	9,516	5,009	3,217	1,290	NR	NR	NR	IOM guidelines	Preeclampsia
Vivatkusol (28)	2017	retrospective study	Thailand	1,943	559	744	640	17.5	17.5	17.3	IOM guidelines	1. C-section 2. Preeclampsia 3. Preterm
Zhang (29)	2020	retrospective study	China	722	230	323	169	NR	NR	NR	IOM guidelines	1. Preeclampsia 2. Cesarean section 3. Macrosomia 4. SGA 5. LGA
Zhang (30)	2022	Prospective Cohort Study	China	31,743	8,608	14,199	8,936	NR	NR	NR	IOM guidelines	Preeclampsia
Omani-Samani (31)	2018	cross-sectional study	Iran	2,394	800	747	847	28.31	28.78	28.77	IOM guidelines	1. Cesarean 2. Preterm birth
Sámano (32)	2018	prospective cohort study	México	601	137	210	254	NR	NR	NR	IOM guidelines	1. Preterm 2. SGA
Almasi-Hashiani (33)	2017	cross-sectional study	Iran	2,168	1,206	645	317	NR	NR	NR	IOM guidelines	1. Cesareana 2. Preterm birth
Yin (34)	2023	retrospective cohort study	China	30,910	10,253	13,088	7,569	30.67 ± 4.31	31.08 ± 4.20	31.68 ± 4.27	ACOG 2013	1. Macrosomia 2. SGA 3. LGA 4. Preterm birth

IOM, Institute of Medicine; SGA, Small-for-gestational-age; LGA, Large-for-gestational-age; WHO, World Health Organization; C-section, Cesarean Section; P-CS, Primary Cesarean Sections.

### 3.3 Quality assessment

The risk of bias in the 17 studies included was evaluated using the NOS. Among the included studies, 15 were cohort studies (17, 19–30, 32, 34), and two were cross-sectional studies (31, 33). Of these, 12 studies received scores of 7–9 stars, indicating high quality, and five studies received a score of 6 stars, indicating moderate quality. The main reasons for the moderate quality included the use of self-reported data for some exposures, lack of control for other important confounding factors, and incomplete follow-up of outcomes. For example, in the study by Monteiro, (19)., exposure was determined using self-reported data (pre-pregnancy BMI was calculated based on self-reported pre-pregnancy weight and height), and there was potential selection bias due to the representativeness of cases (being a multi-center study in Portugal, with most participants being Caucasian from the Mediterranean region, limiting the generalizability to other populations). Additionally, this study did not control for other important confounding factors. In the study by Liu (23), exposure was also determined by self-reporting (pre-pregnancy BMI was calculated based on self-reported pre-pregnancy weight and height, which may lead to reporting bias), and the study did not control for other important confounders (such as dietary and physical activity assessment), and there was incomplete outcome follow-up. Overall, the quality of the included studies was high.

### 3.4 Meta-analysis results of included studies

#### 3.4.1 Cesarean section

Ten studies reported the relationship between GWG and cesarean section. One study (24) focused only on primary cesarean sections (*P*-CS), which occur for reasons such as abnormal fetal position, fetal distress, or macrosomia, excluding repeat cesarean sections. The other nine studies did not differentiate between types of cesarean sections, including elective cesarean sections, emergency cesarean sections before labor, and emergency cesarean sections during labor, regardless of whether vaginal delivery or cesarean section had been performed previously. The total sample size of these 10 studies was 776,594 women. Among them, six studies adjusted for factors such as maternal age, height, smoking, alcohol consumption, socioeconomic status, gestational age, pre-pregnancy BMI, and birth weight, reporting adjusted odds ratios (OR), while four studies did not report the adjusted OR.

**Figure 2:**
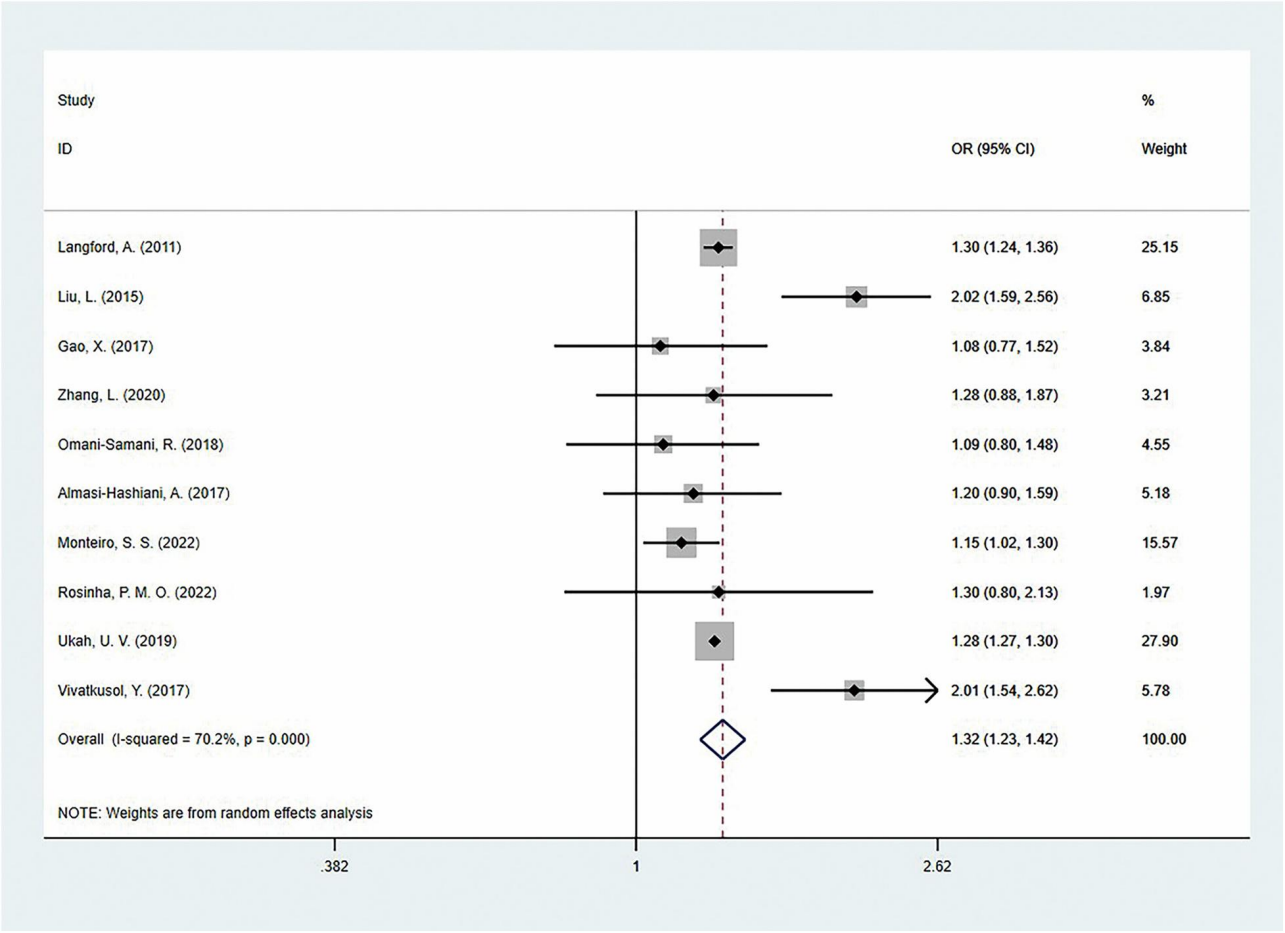
The association of excess gestational weight gain and cesarean section.

The pooled analysis showed that compared to the adequate GWG group, excessive GWG significantly increased the risk of cesarean section [OR = 1.32 (95%CI:1.23–1.42)], but there was moderate heterogeneity between studies (I^2^ = 70.2%) (Figure 2). It is noteworthy that this association varied across different regional populations: the risk increase was greatest among Asian women [OR = 1.41 (95%CI:1.10–1.81)], followed by Europeans [OR = 1.16 (95%CI:1.03–1.31)], and the effect size was relatively smaller in North America [OR = 1.28 (95%CI:1.27–1.30)]. Grouped by national development level, the study found that developing countries had the highest increase in risk [OR = 1.41 (95%CI: 1.10–1.81)], followed by developed countries [OR = 1.28 (95%CI: 1.26–1.30)]. Further analysis based on GWG reference standards revealed that in studies using the IOM guidelines (9 studies), the risk effect of excessive GWG was more pronounced [OR = 1.35 (95%CI 1.18–1.54)], while the only study using WHO standards showed a similar trend [OR = 1.28 (95%CI 1.27–1.30)]. Furthermore, when considering only the adjusted OR, the results still showed that the risk of cesarean section was significantly higher in the excessive GWG group [aOR = 1.32 (95%CI:1.11–1.56); I^2^ = 68.3%; *P* = 0.007] (Table 2).

**Table 2:** Subgroup analysis of the association between the gestational weight gain and cesarean section.

Excess gestational weight gain					
Subgroup	NO. of studies	Sample size	OR (95%CI)	Heterogeneity	
				I^2^(%)	P
Region					
North America	2	756,982	1.28[1.27–1.30]	0%	0.609
Asia	6	11,116	1.41[1.10–1.81]	76.5%	0.001
Europe	2	8,493	1.16[1.03–1.31]	0%	0.646
Level of national development					
Developed country	4	379,085	1.28[1.26–1.30]	6.3%	0.361
Developing country	6	4,779	1.41[1.10–1.81]	76.5%	0.001
Reference standards					
IOM	9	53,755	1.35[1.18–1.54]	73.1%	0.000
OR, Odds Ratios; IOM, Institute of Medicine; WHO, World Health Organization WHO	1	722,839	1.28[1.27–1.30]	-	-
Adjusted					
Adjusted OR only	6	43,319	1.32[1.11–1.56]	68.3%	0.007
Inadequate gestational weight gain					
Region					
North America	2	756,982	0.97[0.90–1.05]	64.1%	0.095
Asia	6	11,116	0.87[0.76–0.99]	0%	0.516
Europe	2	8,493	0.81[0.72–0.90]	0%	0.934
Level of national development					
Developed country	4	148,831	0.91[0.81–1.03]	82.2%	0.001
Developing country	6	2,548	0.87[0.76–0.99]	0%	0.516
Reference standards					
IOM	9	53,755	0.87[0.81–0.93]	0%	0.517
WHO	1	722,839	1.00[0.99–1.01]	-	-
Adjusted					
Adjusted OR only	6	43,319	0.90[0.83–0.98]	0%	0.448

OR, Odds Ratios; IOM, Institute of Medicine; WHO, World Health Organization.

**Figure 3:**
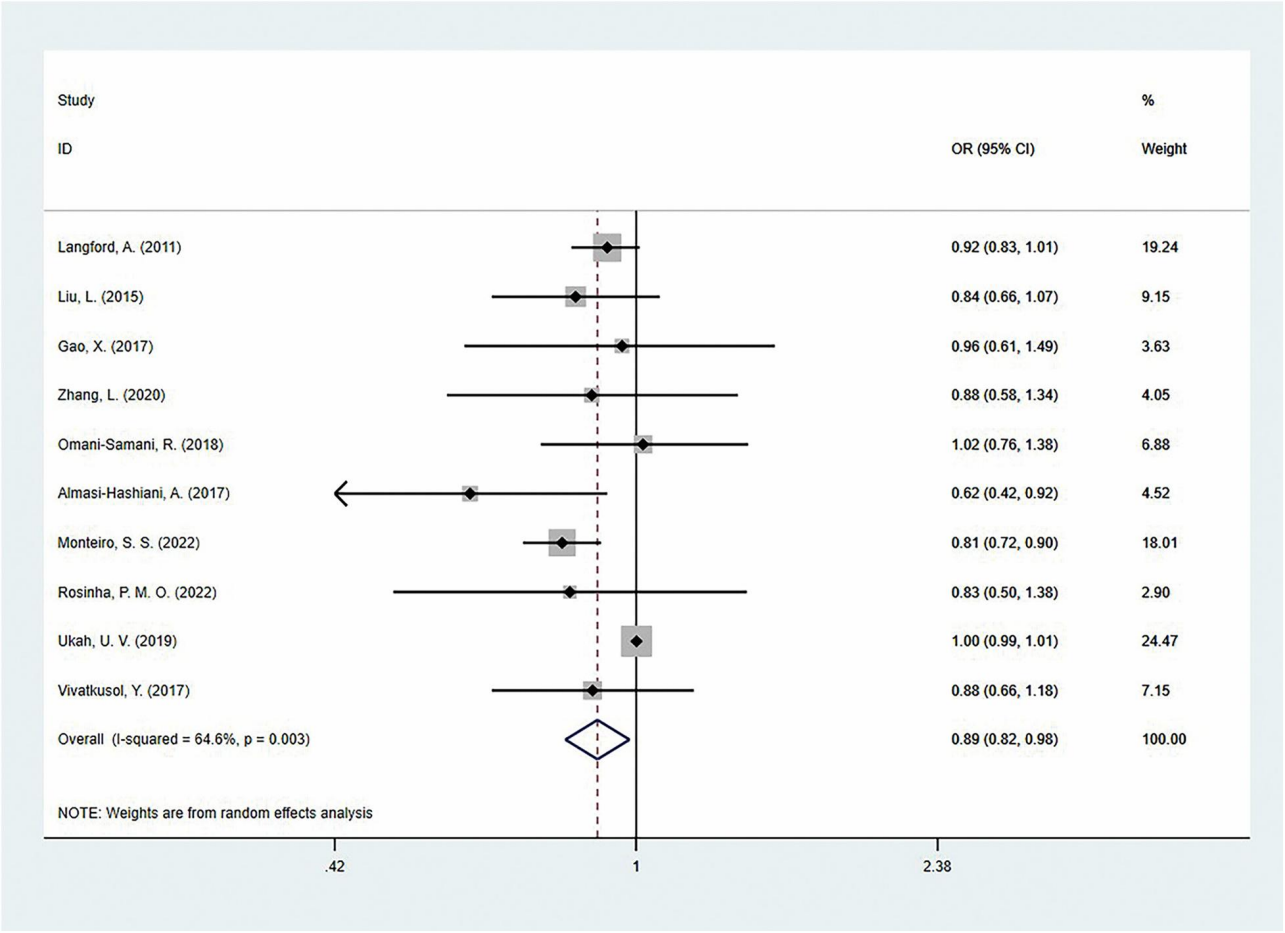
The association of inadequate gestational weight gain and cesarean section.

Conversely, insufficient GWG exhibited a protective effect, generally reducing the cesarean section risk by 11% [OR = 0.89 (95%CI:0.82–0.98); *I*^2^ = 64.6%, *P* = 0.003] (Figure 3). This protective effect was particularly evident in Asian [OR = 0.87 (95%CI:0.76–0.99)] and European populations [OR = 0.81 (95%CI:0.72–0.90)], but it was not statistically significant in North America [OR = 0.97 (95%CI:0.90–1.05)]. In the analysis across countries with different development levels, developing countries showed a significantly reduced risk of cesarean section [OR =  0.87 (95%CI: 0.76–0.99); *I*^2^ = 0.0%, *P* = 0.516], while no statistically significant difference was observed in developed countries [OR = 0.91 (95%CI: 0.81–1.03)]. Under the IOM guidelines, insufficient GWG remained associated with a reduced risk of cesarean section, with no significant heterogeneity between studies [OR = 0.87 (95%CI:0.81–0.93), *I*^2^ = 0%, *P* = 0.517], while no significant association was found under the WHO standards. Notably, 6 studies (60%) provided adjusted ORs, and the pooled results showed that insufficient gestational weight gain reduced the risk of cesarean section [OR = 0.90 (95%CI:0.83–0.98); *I*^2^ = 0.0%; *P* = 0.448] (Table 2).

#### 3.4.2 Preterm birth

Nine studies reported the relationship between GWG and preterm birth. Among these, five studies adjusted for factors such as maternal age, height, socioeconomic status, number of previous pregnancies, history of preeclampsia, family history of hypertension and diabetes, pre-pregnancy BMI, cesarean section history, and birth weight, reporting adjusted OR values. The remaining four studies did not report adjusted OR values. The total sample sizes for the excessive GWG group, adequate GWG group, and insufficient GWG group were 17,066, 19,233, and 13,625, respectively. Significant heterogeneity was observed across studies, and a random-effects model was used to combine the data.

**Figure 4:**
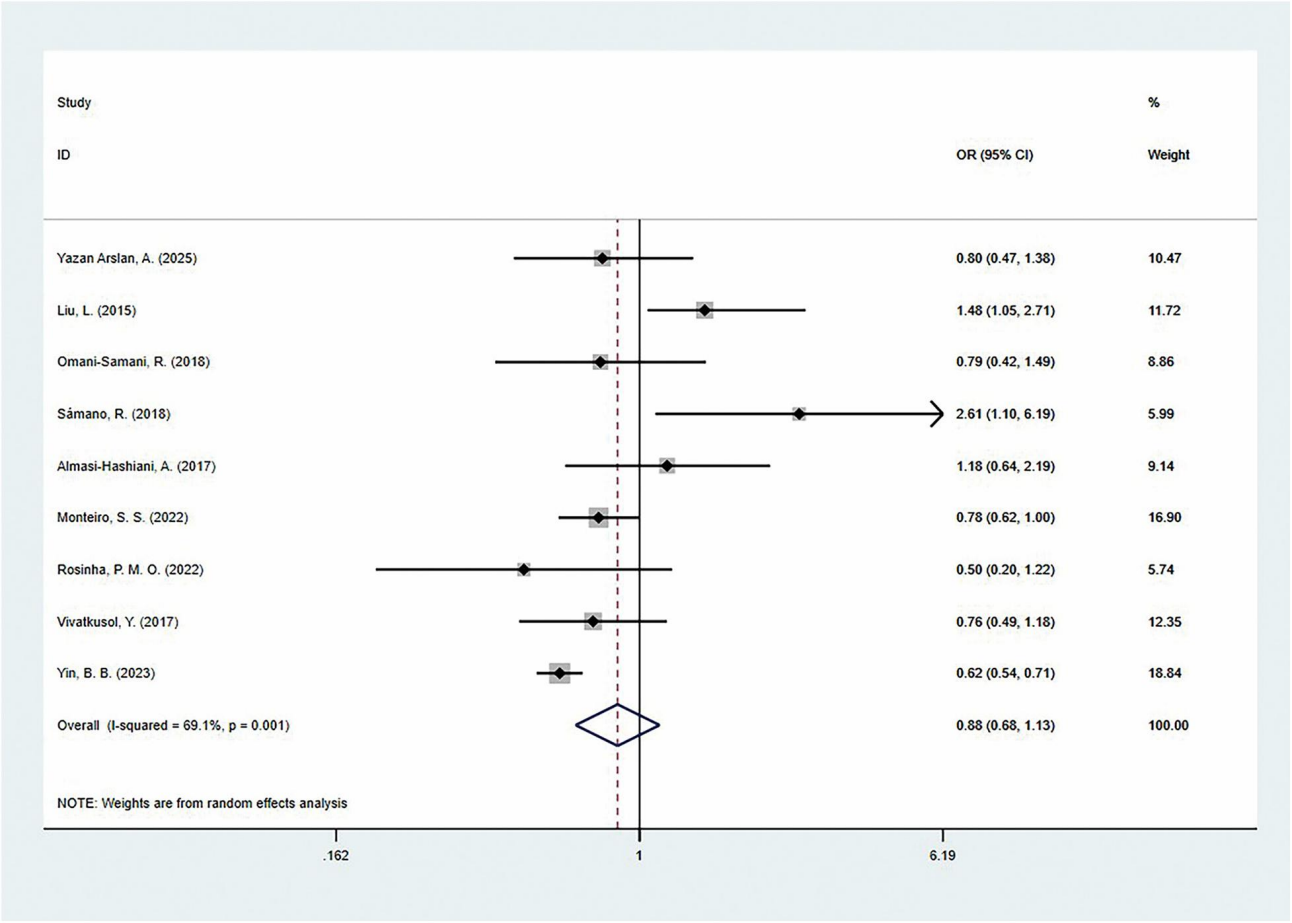
The association of excess gestational weight gain and preterm delivery.

The pooled analysis showed that, compared to the adequate GWG group, excessive GWG was not significantly associated with preterm birth [OR = 0.88 (95% CI: 0.68–1.13)], but there was moderate heterogeneity (*I*^2^ = 69.1%) (Figure 4). Notably, this association varied across different regional populations: North American women may show the highest risk increase [OR = 2.61 (95% CI: 1.10–6.19)], while European women showed a significant reduction in the risk of preterm birth [OR = 0.76 (95% CI: 0.60–0.96)], and the association was not statistically significant in Asian women [OR = 0.86 (95% CI: 0.63–1.18)]. When stratified by national development level, preterm birth risk was found to be reduced by 24% in developed countries [OR = 0.76 (95% CI: 0.60–0.96)], whereas no association was observed in developing countries [OR = 0.97 (95% CI: 0.68–1.37)]. Further analysis by GWG reference standard revealed that the only study using the ACOG 2013 standard showed a protective effect for excessive GWG [OR = 0.62 (95% CI: 0.54–0.71)], while studies using the IOM guidelines (8 studies) did not show a significant association [OR = 0.95 (95% CI: 0.72–1.24)]. Moreover, when only the adjusted ORs were summarized, the results showed that this association remained non-significant [OR = 1.17 (95% CI: 0.81–1.71)] (Table 3).

**Table 3:** Subgroup analysis of the association between the gestational weight gain and preterm birth.

Subgroup	NO. of studies	Sample size	OR (95%CI)	Heterogeneity	
				I^2^(%)	P
Excess gestational weight gain					
Region					
Asia	6	41,030	0.86[0.63–1.18]	68.5%	0.007
North America	1	601	2.61[1.10–6.19]	-	-
Europe	2	8,493	0.76[0.60–0.96]	0%	0.340
Level of national development					
Developed country	2	2,319	0.76[0.60–0.96]	0%	0.340
Developing country	7	14,791	0.97[0.68–1.37]	75.8%	0.000
Reference standards					
IOM	8	19,214	0.95[0.72–1.24]	52.5%	0.040
ACOG2013	1	30,910	0.62[0.54–0.71]	-	-
Adjusted					
Adjusted OR only	5	8,778	1.17[0.81–1.71]	47.7%	0.105
Inadequate gestational weight gain					
Region					
Asia	6	41,030	1.54[1.14–2.09]	74.8%	0.001
North America	1	601	2.61[1.09–6.26]	-	-
Europe	2	8,493	1.06[0.87–1.28]	0%	0.876
Level of national development					
Developed country	2	3,535	1.06[0.87–1.28]	0%	0.876
Developing country	7	10,204	1.60[1.21–2.13]	70.4%	0.002
Reference standards					
IOM	8	19,214	1.29[1.05–1.58]	36.7%	0.136
ACOG2013	1	30,910	2.12[1.90–2.37]	-	-
Adjusted					
Adjusted OR only	5	8,778	1.39[1.01–1.92]	35.3%	0.186

OR, Odds Ratios; IOM, Institute of Medicine; ACOG, American College of Obstetricians and Gynecologists.

**Figure 5:**
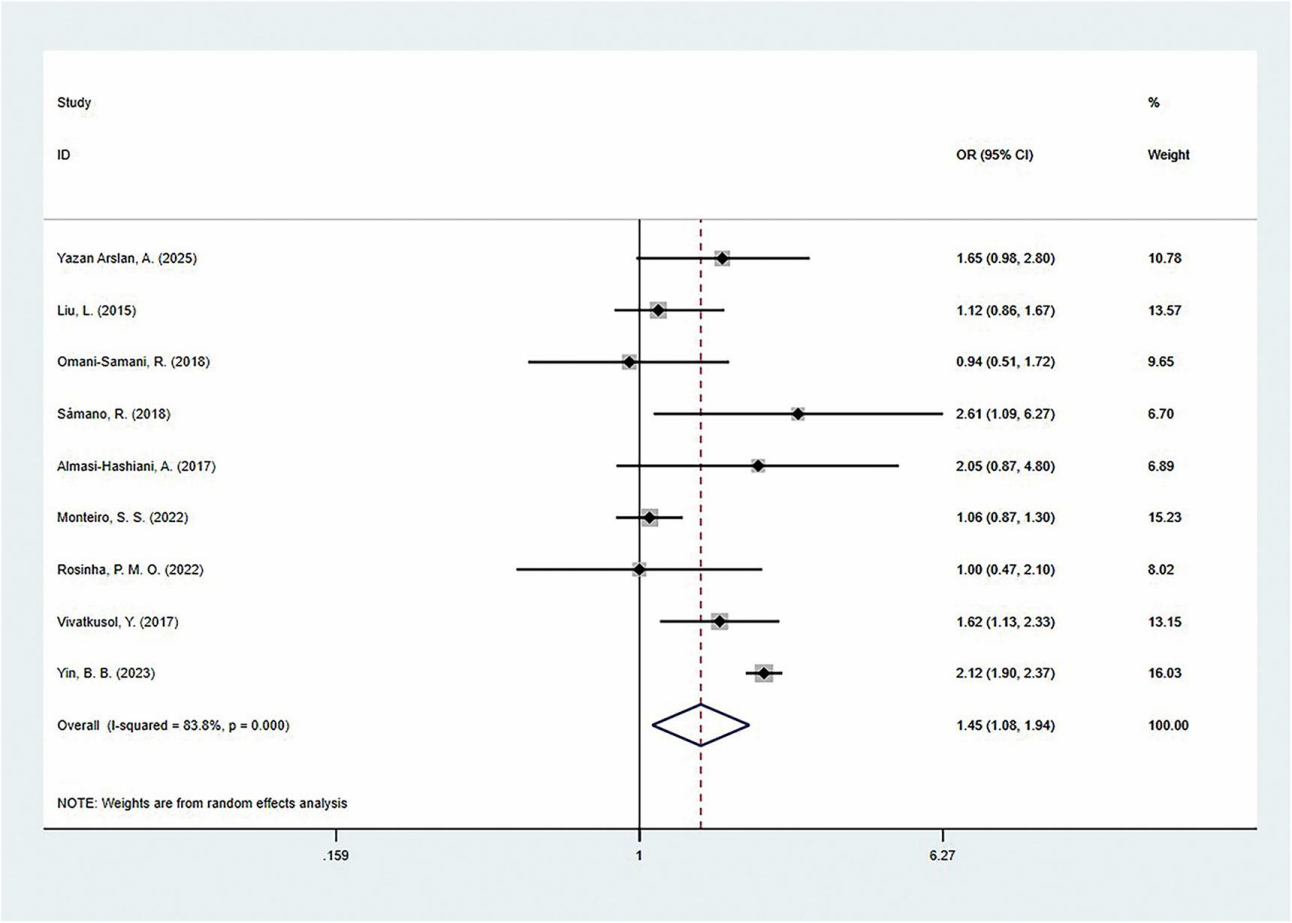
The association of inadequate gestational weight gain and preterm delivery.

In contrast, insufficient GWG significantly increased the risk of preterm birth [OR = 1.45 (95% CI: 1.08–1.94)], but there was high heterogeneity across studies (*I*^2^ = 83.8%) (Figure 5). This association also varied significantly across regional populations: North American women had the largest risk increase [OR = 2.61 (95% CI: 1.09–6.26)], followed by Asian women [OR = 1.54 (95% CI: 1.14–2.09)], while European women showed no significant association [OR = 1.06 (95% CI: 0.87–1.28)]. In addition, we found that the risk was more significant in developing countries [OR = 1.60 (95%CI: 1.21–2.13)], while no significant difference was observed in developed countries [OR = 1.06 (95%CI: 0.87–1.28)]. Further analysis by GWG reference standard showed that the only study using the ACOG 2013 standard showed a more prominent risk effect [OR = 2.12 (95% CI: 1.90–2.37)], while studies using the IOM guidelines (8 studies) also showed a similar trend [OR = 1.29 (95% CI: 1.05–1.58)]. Additionally, when only the adjusted ORs were summarized, the results still showed a significant increase in the risk of preterm birth [OR = 1.39 (95% CI: 1.01–1.92)] (Table 3).

#### 3.4.3 Preeclampsia

A total of eight studies reported the relationship between GWG and preeclampsia, all of which used the IOM guidelines as the GWG reference standard. Among these, five studies adjusted for factors such as maternal age, education level, household income, residential area, height, smoking, alcohol consumption, gestational age, family history of hypertension, family history of diabetes, and pre-pregnancy BMI, reporting adjusted OR values, while three studies did not report adjusted OR values.

**Figure 6:**
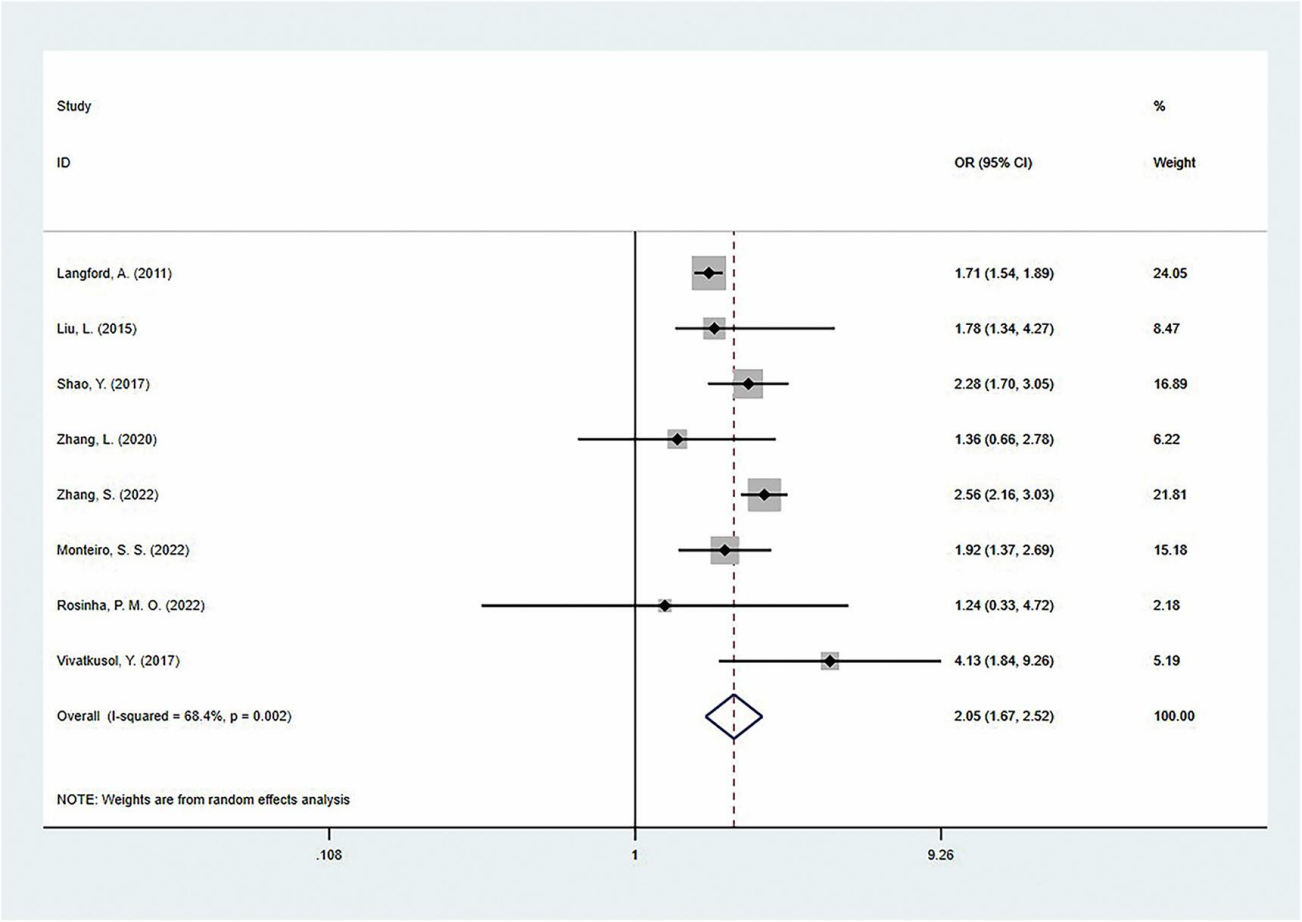
The association of excess gestational weight gain and preeclampsia.

The pooled analysis showed that, compared to the adequate GWG group, excessive GWG significantly increased the risk of preeclampsia [OR = 2.05 (95% CI: 1.67–2.52)], but there was moderate heterogeneity between studies (*I*^2^ = 68.4%) (Figure 6). Notably, this association varied across different regional populations: Asian women showed the highest risk increase [OR = 2.34 (95% CI: 1.89–2.88)], followed by European women [OR = 1.87 (95% CI: 1.35–2.59)], while North American women showed a relatively smaller effect size [OR = 1.71 (95% CI: 1.54–1.89)]. In subgroup analyses based on differences in national development levels, a similar relationship was observed in both developing countries [OR = 2.34 (95%CI: 1.89–2.88)] and developed countries [OR = 1.72 (95%CI: 1.56–1.90)]. Moreover, when only the adjusted ORs were summarized, the results showed a similar trend for excessive GWG [OR = 2.01 (95% CI: 1.57–2.57); *I*^2^ = 78.1%; *P* = 0.001] (Table 4).

**Table 4:** Subgroup analysis of the association between the gestational weight gain and preeclampsia.

Subgroup	NO. of studies	Sample size	OR (95%CI)	Heterogeneity	
				I^2^(%)	P
Excess gestational weight gain					
Region					
North America	1	34,143	1.71[1.54–1.89]	-	-
Asia	5	46,897	2.34[1.89–2.88]	31.1%	0.214
Europe	2	8,493	1.87[1.35–2.59]	0%	0.534
Level of national development					
Developed country	3	27,470	1.72[1.56–1.90]	0%	0.721
Developing country	5	16,006	2.34[1.89–2.88]	31.1%	0.214
Adjusted					
Adjusted OR only	5	79,097	2.01[1.57–2.57]	78.1%	0.001
Inadequate gestational weight gain					
Region					
North America	1	34,143	0.78[0.61–0.99]	-	-
Asia	5	46,897	1.15[0.71–1.85]	85.1%	0.000
Europe	2	8,493	0.81[0.57–1.16]	0%	0.377
Level of national development					
Developed country	3	5,322	0.79[0.65–0.97]	0%	0.664
Developing country	5	11,439	1.15[0.71–1.85]	85.1%	0.000
Adjusted					
Adjusted OR only	5	79,097	1.13[0.69–1.86]	89.0%	0.000

OR, Odds Ratios.

**Figure 7:**
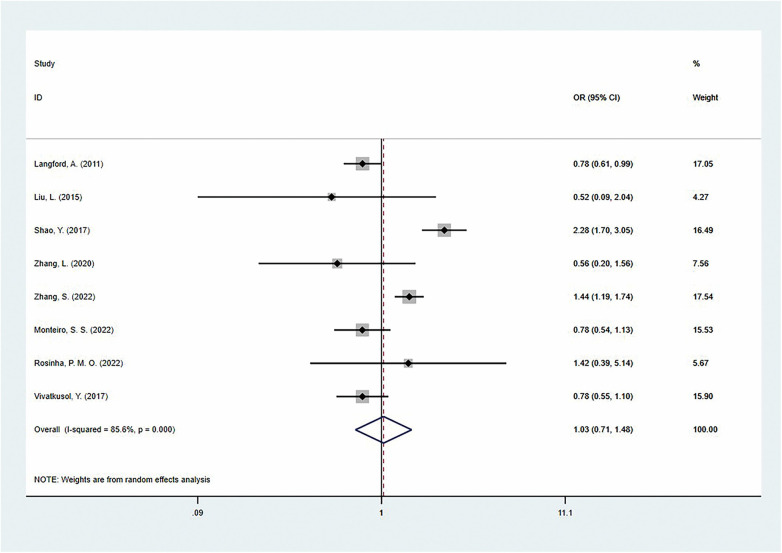
The association of inadequate gestational weight gain and preeclampsia.

Conversely, insufficient GWG did not show a statistically significant association with preeclampsia [OR = 1.03 (95% CI: 0.71–1.48); *I*^2^ = 85.6%; *P* = 0.000] (Figure 7). However, further analysis revealed a protective effect in North American women [OR = 0.78 (95% CI: 0.61–0.99)], while no statistically significant association was found in Asian or European women. Subgroup analysis based on national development levels revealed that in developed countries, women with insufficient gestational weight gain also had a significantly reduced risk of preeclampsia [OR =  0.79 (95%CI: 0.65–0.97)], while no statistical significance was observed in developing countries [OR = 1.15 (95%CI: 0.71–1.85)]. Notably, 5 studies (62.5%) provided adjusted ORs, and the pooled results showed no significant association [OR = 1.13 (95% CI: 0.69–1.86); *I*^2^ = 89.0%; *P* = 0.000] (Table 4).

#### 3.4.4 Macrosomia

A total of eight studies reported the relationship between excessive GWG and macrosomia, of which seven studies used the IOM guidelines as the GWG reference standard and one study used the ACOG 2013 guidelines. Among these, four studies adjusted for factors such as maternal age, height, number of pregnancies, gestational age at delivery, family history of diabetes, and pre-pregnancy BMI, reporting adjusted OR values, while four studies did not report adjusted OR values. Our findings indicate that excessive GWG significantly increased the risk of macrosomia [OR = 2.18 (95% CI: 2.03–2.35); *I*^2^ = 0.0%; *P* = 0.875]. On the other hand, insufficient GWG decreased the risk of macrosomia [OR = 0.70 (95% CI: 0.55–0.91); *I*^2^ = 69.4%; *P* = 0.002].

#### 3.4.5 Large for gestational age (LGA)

Seven studies reported the relationship between GWG and LGA. Among these, four studies adjusted for factors such as maternal age, education level, smoking, alcohol consumption, family income, number of pregnancies, pre-pregnancy BMI, and infant gender, reporting adjusted OR values, while three studies did not report adjusted OR values. There was no significant heterogeneity across the studies in the excessive GWG group, and a fixed-effects model was used to combine the data. Our findings showed that excessive GWG significantly increased the risk of LGA [OR = 2.20 (95% CI: 1.97–2.46); *I*^2^ = 0.0%; *P* = 0.680]. Conversely, insufficient GWG decreased the risk of LGA [OR = 0.65 (95% CI: 0.56–0.75); *I*^2^ = 0.0%; *P* = 0.712].

#### 3.4.6 Small for gestational age (SGA)

Five studies reported the relationship between GWG and SGA, all of which were published in the last 10 years. Among these, four studies used the IOM guidelines as the GWG reference standard, and one study used the ACOG 2013 guidelines. Four studies adjusted for factors such as maternal age, education level, smoking, alcohol consumption, number of pregnancies, pre-pregnancy BMI, and infant gender, reporting adjusted OR values, while one study did not report adjusted OR values. There was significant heterogeneity across studies, and a random-effects model was used to combine the data. Our findings showed that both excessive and insufficient GWG were not significantly associated with SGA.

### 3.5 Publication bias and sensitivity analysis

**Figure 8:**
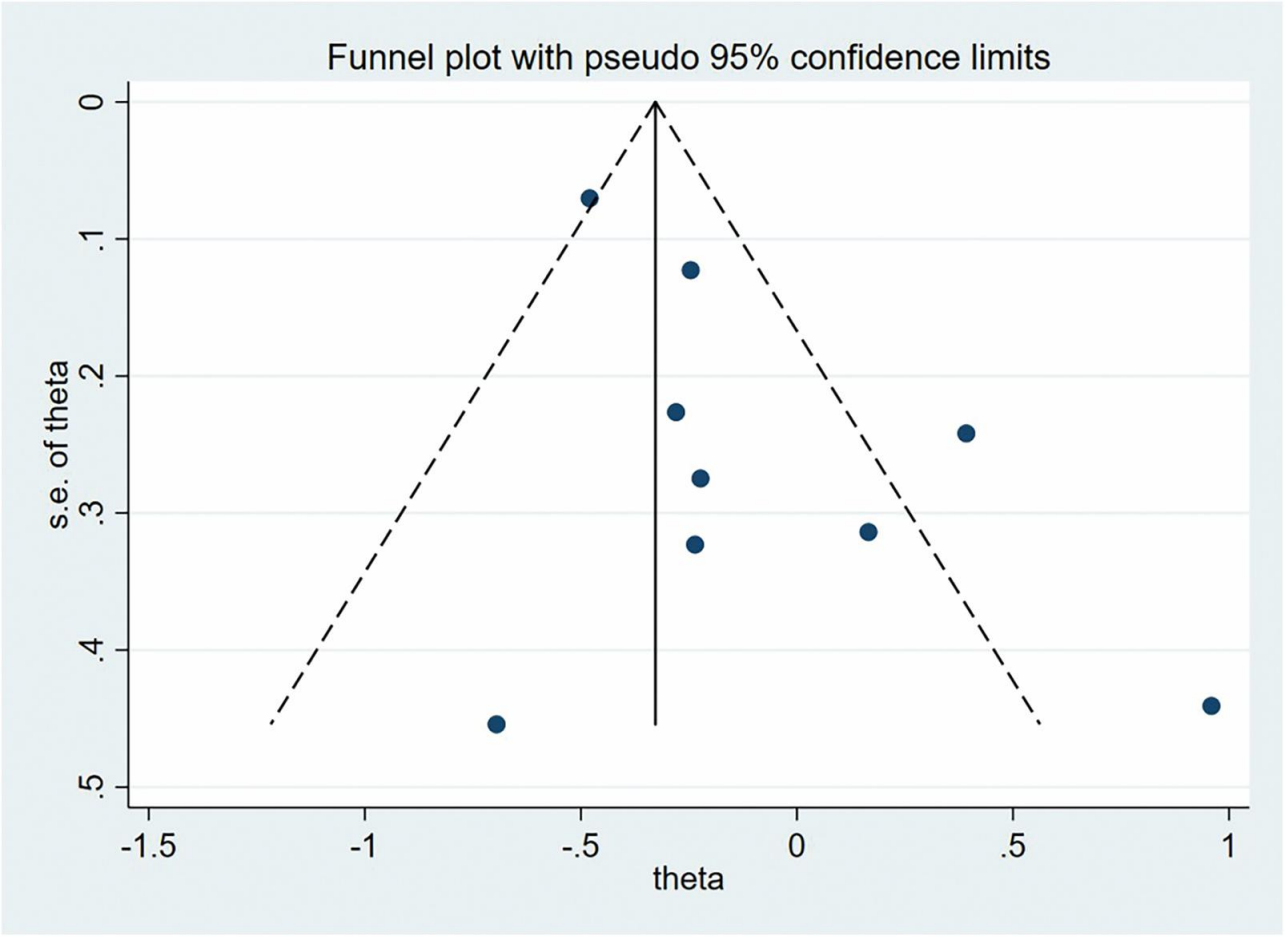
Funnel plot of the effect of excessive weight gain during pregnancy on preterm birth.

We conducted publication bias analysis for the included indicators using funnel plots to visually assess publication bias. Additionally, Egger's test was used to analyze the symmetry of the funnel plots, with *P* > 0.05 indicating no significant publication bias (Figure 8). Specifically, in the relationship between insufficient GWG and cesarean section, the Egger's test result was *P* = 0.018, suggesting potential publication bias. We applied the trim-and-fill method to adjust for publication bias. The results showed that no potential missing studies were identified, and after adjustment, the combined effect size remained unchanged, indicating that publication bias did not significantly impact the meta-analysis results.

**Figure 9:**
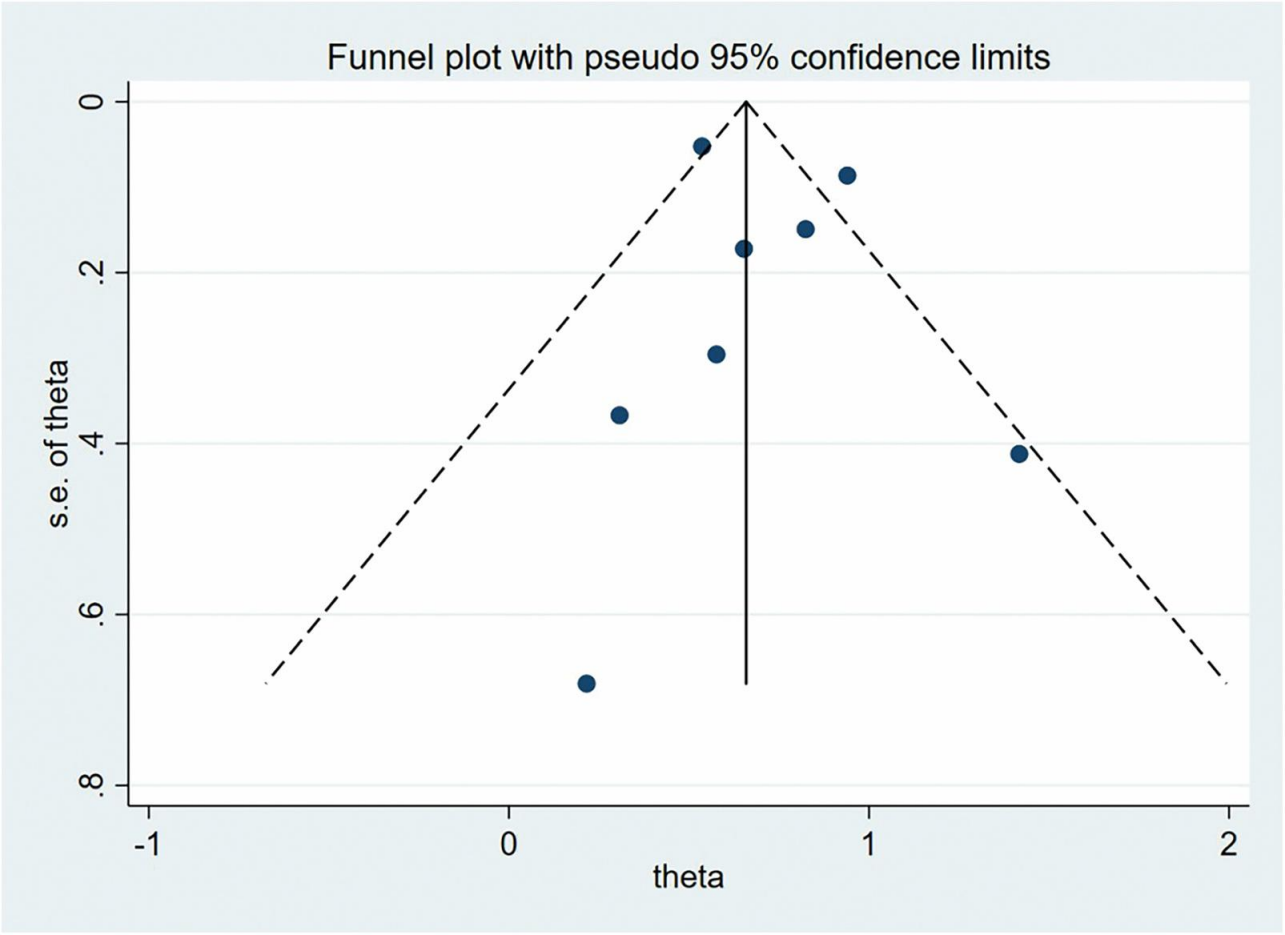
Funnel plot of the effect of excessive gestational weight gain on preeclampsia.

For sensitivity analysis, we used the leave-one-out method. The results showed that excluding any individual study did not lead to significant changes in the combined effect size, indicating that the meta-analysis results were robust and not excessively influenced by any single study (Figure 9). The complete funnel plots, publication bias, and sensitivity analysis results are provided in the supplementary materials.

## 4 Discussion

This study integrated data from 17 global cohort studies, comprising 866,593 women, to systematically assess the relationship between GWG and adverse maternal and infant outcomes. The results showed that among the included population, 47.84% of women exceeded the recommended GWG range, 19.98% did not meet the guidelines, and only 32.18% were within the recommended range. The meta-analysis indicated that excessive GWG significantly increases the risk of cesarean section, preeclampsia, macrosomia, and LGA, while there was no statistically significant association with preterm birth and SGA. In contrast, insufficient GWG was associated with a higher risk of preterm birth but a lower risk of cesarean section, macrosomia, and LGA, with no significant relationship to preeclampsia or SGA.

In line with the study by Goldstein RF et al. (35) and that of Victor A et al. (36).our findings also found that both excessive and insufficient GWG, compared to the recommended guidelines, were linked to an increased risk of adverse maternal and infant outcomes. However, Goldstein RF et al.'s study (35) lacked cohorts from developing countries, whereas our study includes data from such regions. The study results also showed that for cesarean section, excessive GWG demonstrated a stronger risk-increasing effect in developing countries (OR = 1.41), while insufficient GWG only exhibited a protective effect in developing countries (OR = 0.87). For preterm birth, excessive GWG reduced the risk in developed countries (OR = 0.76) but showed no association in developing countries; insufficient GWG significantly increased the risk in developing countries (OR = 1.60) but had no association in developed countries. For preeclampsia, excessive GWG showed the greatest risk increase in developing countries (OR = 2.34), while insufficient GWG exhibited a protective effect in developed countries (OR = 0.79) but no association in developing countries. Additionally, our study uniquely addressed the relationship between GWG and preeclampsia, based on 8 studies (18, 19, 22–30). We also clearly identified that excessive GWG significantly increases the risk of preeclampsia, this is consistent with the conclusion of the research by Filipe Dias de Souza et al. (37). With subgroup analysis revealing that Asian women in the excessive GWG group had the highest risk. In contrast, Asefa F et al.'s study (38) found no significant difference in the risk of preeclampsia between women with insufficient or adequate GWG, and no significant correlation between excessive GWG and macrosomia. Our study, however, found that excessive GWG is associated with both an increased risk of preeclampsia and macrosomia. These discrepancies may be attributed to differences in the racial, economic, and pre-pregnancy characteristics of the populations studied, as well as differences in metabolic states among the mothers. Therefore, weight management during pregnancy is crucial in reducing the rates of cesarean section, preeclampsia, and macrosomia. Our findings are particularly relevant for use in developing countries, with caution. Additionally, in contrast to previous studies, we innovatively conducted subgroup analyses based on different GWG reference standards, revealing the impact of these standards on effect sizes. Due to ethical constraints, researchers are often unable to conduct adequate clinical trials (as excessive weight gain during pregnancy may negatively affect maternal and infant health). Consequently, the most adequate and highest-level study design for these clinical issues is cohort studies. By conducting a quality assessment of the included cohort studies and performing a pooled analysis, we incorporated data from over 860,000 women globally, providing substantial data support for the study. Moreover, this study excluded research published before the 2009 IOM guidelines, avoiding historical bias.

From a pathophysiological perspective, excessive weight gain during pregnancy leads to increased fat accumulation in the body. The additional fat produces more fatty acids, which cause insulin resistance and increase peripheral vascular resistance. This insulin resistance drives hyperglycemia and dyslipidemia, while concurrently increasing sympathetic tone and peripheral vascular resistance, thereby promoting endothelial dysfunction and hypertension—a central pathway in preeclampsia development (39). Additionally, excessive weight gain during pregnancy leads to the accumulation of fat in the maternal tissues, some of which accumulate in the birth canal, narrowing it and causing labor dystocia, thereby increasing the cesarean section rate. On the other hand, insufficient weight gain during pregnancy directly leads to depletion of the mother's macronutrient and micronutrient reserves, disrupting the supply of energy substrates (such as glucose and amino acids) to the fetus and inhibiting protein synthesis and organ development. Additionally, micronutrient deficiencies—especially iron and folate—induce maternal and fetal anemia, damage placental vascular endothelium, and elevate homocysteine levels, which inhibit nitric oxide bioavailability and further impair placental perfusion and oxygen diffusion capacity (40, 41). From an endocrine perspective, insufficient weight gain in pregnant women can also result in maternal malnutrition, which limits trophoblast invasion and causes spiral artery remodeling failure. This increases placental vascular resistance and reduces the surface area for villous exchange (on average, by 22%), leading to chronic fetal hypoxia and reduced nutrient transport efficiency. This significantly increases the risk of SGA and low birth weight (LBW) (42).

The regional variations observed in our meta-analysis may be partially explained by differences in cultural perceptions of pregnancy and healthcare infrastructure. In certain cultures, the traditional belief of ‘eat more” remains prevalent, which may inadvertently encourage excessive GWG. Conversely, in other societies, a strong emphasis on maternal slimness might lead to inadequate gain. Furthermore, the capacity of healthcare systems to provide consistent prenatal monitoring and professional nutritional counseling varies significantly. In developed nations, adherence to guidelines like the IOM is often supported by integrated care pathways. In contrast, in some developing regions, limited access to regular prenatal visits and a lack of standardized weight-monitoring protocols may exacerbate the risks associated with abnormal GWG. Other unmeasured confounders, such as maternal socioeconomic status, educational attainment, and local dietary patterns, also play a critical role in shaping the relationship between weight gain and pregnancy outcomes.

Despite providing important evidence on the relationship between GWG and pregnancy outcomes through a rigorous methodological design, this study should be interpreted with caution due to several limitations. First, the geographic distribution of the data is uneven, with more than 80% of the data coming from Asia and North America, and a lack of data from other regions of the world. This limits the generalizability of the conclusions. Second, different studies did not consistently define preterm birth and cesarean section. For example, there was no clear distinction between spontaneous and medically induced preterm birth, or between emergency and elective cesarean sections, nor between primary and repeat cesarean sections. This inconsistency limits the interpretation of the results. Moreover, some studies assessed exposure through self-reporting rather than direct measurements by healthcare professionals. Specifically, this included recording the last known weight before pregnancy, rather than measuring the weight at the first prenatal visit, which may introduce additional errors. Due to the lack of detailed data in the original included studies, we were unable to fully account for certain confounding factors, such as gestational diabetes or pre-existing diabetes, chronic hypertension, and assisted reproductive technologies. These conditions are known to influence both maternal weight gain patterns and the risk of adverse pregnancy outcomes. Although most included studies adjusted for basic confounders like maternal age and pre-pregnancy BMI, the potential for residual confounding remains. Future primary research should provide more detailed participant characteristics to allow for more nuanced analyses. Finally, the results of this study are based on observational data, which cannot establish causal relationships.

These limitations reflect the common challenges in current pregnancy nutrition research and provide direction for future studies, it is expected that future research can take into account confounding factors such as gestational diabetes or pre-existing diabetes, chronic hypertension, assisted reproductive technologies, twins and multiple births. Future research should be global in scope and carried out through multidisciplinary collaborations, with more high-quality randomized controlled trials. Additionally, standardized GWG measurement protocols should be established, with a focus on exploring personalized GWG management strategies based on pre-pregnancy BMI and metabolic characteristics.

## 5 Conclusion

Abnormal gestational weight gain is significantly associated with adverse pregnancy outcomes, with effects that vary by population context. This meta-analysis of 17 multinational cohort studies demonstrates that excessive gestational weight gain increases the risks of cesarean delivery, preeclampsia, macrosomia, and large-for-gestational-age infants.

Notably, these associations differed across geographic regions, levels of national development, and guideline standards, highlighting the context-dependent nature of gestational weight gain–related risks. These findings suggest that uniform gestational weight gain recommendations may not be universally adequate and support the need for population-specific and individualized gestational weight management strategies, particularly in developing countries.

## Data Availability

The datasets supporting the conclusions of this article are included within the article and its supplementary files.
