# Supplementary material for: Association between gestational weight gain and adverse pregnancy outcomes: a systematic review and meta-analysis
Source: Front Glob Womens Health. 2026 Apr 28;7:1777069. doi: 10.3389/fgwh.2026.1777069 (PMC13161064; doi:10.3389/fgwh.2026.1777069)
Supplement: Supplementary file 1 [file Table1.docx]

Supplementary Material

**Table S1.** Detailed search strategy of PubMed

| Search | Query | n1u  **Items** |
| --- | --- | --- |
| #1 | Search (((((Gestational Weight Gain[MeSH Terms]) ) OR (Gestational Weight Gain[Title/Abstract])) OR (Maternal Weight Gain[Title/Abstract])) OR (Pregnancy Weight Gain[Title/Abstract])) OR (Postpartum Weight Retention[Title/Abstract]) | 6668 |
| #2 | Search(((((pregnancy outcome[MeSH Terms]) OR (pregnancy outcome[MeSH Terms])) OR (Pregnancy Outcome[Title/Abstract])) OR (Pregnancy Outcome*[Title/Abstract])) OR (birth outcome[Title/Abstract])) OR (obstetric outcome[Title/Abstract]) | 110634 |
| #3 | Search(((((((((Pregnancy Complications[MeSH Terms]) OR (Pregnancy Complications[Title/Abstract])) OR (Pregnancy Complications[Title/Abstract])) OR (Adverse Birth Outcome*[Title/Abstract])) OR (complication* during pregnancy[Title/Abstract])) OR (complications related to pregnancy[Title/Abstract])) OR (disease* complicating pregnancy[Title/Abstract])) OR (gestational complication*[Title/Abstract])) OR (pregnancy neoplastic complications[Title/Abstract])) OR (pregnancy-associated complications[Title/Abstract]) | 502009 |
| #4 | Search ((((((pregnancy outcome[MeSH Terms]) OR (pregnancy outcome[MeSH Terms])) OR (Pregnancy Outcome[Title/Abstract])) OR (Pregnancy Outcome*[Title/Abstract])) OR (birth outcome[Title/Abstract])) OR (obstetric outcome[Title/Abstract])) OR ((((((((((Pregnancy Complications[MeSH Terms]) OR (Pregnancy Complications[Title/Abstract])) OR (Pregnancy Complications[Title/Abstract])) OR (Adverse Birth Outcome*[Title/Abstract])) OR (complication* during pregnancy[Title/Abstract])) OR (complications related to pregnancy[Title/Abstract])) OR (disease* complicating pregnancy[Title/Abstract])) OR (gestational complication*[Title/Abstract])) OR (pregnancy neoplastic complications[Title/Abstract])) OR (pregnancy-associated complications[Title/Abstract])) | 537626 |
| #5 | Search ((((((Gestational Weight Gain[MeSH Terms]) ) OR (Gestational Weight Gain[Title/Abstract])) OR (Maternal Weight Gain[Title/Abstract])) OR (Pregnancy Weight Gain[Title/Abstract])) OR (Postpartum Weight Retention[Title/Abstract])) AND (((((((pregnancy outcome[MeSH Terms]) OR (pregnancy outcome[MeSH Terms])) OR (Pregnancy Outcome[Title/Abstract])) OR (Pregnancy Outcome*[Title/Abstract])) OR (birth outcome[Title/Abstract])) OR (obstetric outcome[Title/Abstract])) OR ((((((((((Pregnancy Complications[MeSH Terms]) OR (Pregnancy Complications[Title/Abstract])) OR (Pregnancy Complications[Title/Abstract])) OR (Adverse Birth Outcome*[Title/Abstract])) OR (complication* during pregnancy[Title/Abstract])) OR (complications related to pregnancy[Title/Abstract])) OR (disease* complicating pregnancy[Title/Abstract])) OR (gestational complication*[Title/Abstract])) OR (pregnancy neoplastic complications[Title/Abstract])) OR (pregnancy-associated complications[Title/Abstract]))) | 3971 |

**Table S2.** Detailed search strategy of Embase

| Search | Query | n1u  **Items** |
| --- | --- | --- |
| #1 | Search 'gestational weight gain'/exp | 5905 |
| #2 | Search 'gestational weight gain':ab,ti OR 'maternal weight gain':ab,ti OR 'pregnancy weight gain':ab,ti OR 'postpartum weight retention':ab,ti | 8751 |
| #3 | Add search(#1 OR #2) | 10538 |
| #4 | Search 'pregnancy outcome'/exp | 94159 |
| #5 | Search 'pregnancy outcome':ab,ti OR 'pregnancy outcome*':ab,ti OR 'birth outcome':ab,ti OR 'obstetric outcome':ab,ti | 57525 |
| #6 | Add search(#4 OR #5) | 110228 |
| #7 | Search 'pregnancy complication'/exp | 698051 |
| #8 | Search 'pregnancy complications':ab,ti OR 'pregnancy complication':ab,ti OR 'adverse birth outcome':ab,ti OR 'complication* during pregnancy':ab,ti OR 'complications related to pregnancy':ab,ti OR 'disease* complicating pregnancy':ab,ti OR 'gestational complication*':ab,ti OR 'pregnancy neoplastic complications':ab,ti OR 'pregnancy-associated complications':ab,ti | 18886 |
| #9 | Add search(#7 OR #8) | 701585 |
| #10 | Add search(#6 OR #9) | 748101 |
| #11 | Add search(#3 AND #10) | 6827 |

**Table S3.** Detailed search strategy of Cochrane Library

| Search | Query | n1u  **Items** |
| --- | --- | --- |
| #1 | Search MeSH descriptor: [Gestational Weight Gain] explode all trees | 252 |
| #2 | Search (Gestational Weight Gain):ti,ab,kw OR (Maternal Weight Gain):ti,ab,kw OR (Pregnancy Weight Gain):ti,ab,kw OR (Postpartum Weight Retention):ti,ab,kw | 3342 |
| #3 | Add search(#1 or #2) | 3342 |
| #4 | Search MeSH descriptor: [Pregnancy Outcome] explode all trees | 5100 |
| #5 | Search (pregnancy outcome*):ti,ab,kw OR (birth outcome):ti,ab,kw OR (obstetric outcome):ti,ab,kw | 59996 |
| #6 | Add search(#4 or #5) | 60358 |
| #7 | Search MeSH descriptor: [Pregnancy Complications] explode all trees | 17787 |
| #8 | Search (Pregnancy Complications):ti,ab,kw OR (Adverse Birth Outcome*):ti,ab,kw OR (complication* during pregnancy):ti,ab,kw OR (complications related to pregnancy):ti,ab,kw OR (disease* complicating pregnancy):ti,ab,kw | 19982 |
| #9 | Search (gestational complication*):ti,ab,kw OR (pregnancy neoplastic complications):ti,ab,kw OR (pregnancy-associated complications):ti,ab,kw | 5915 |
| #10 | Add search(#7 or #8 or #9) | 31572 |
| #11 | Add search(#6 or #10) | 72649 |
| #12 | Add search(#3 and #11) | 2226 |

**Table S4.** Detailed search strategy of Web of Science

| Search | Query | n1u  **Items** |
| --- | --- | --- |
| #1 | Search Gestational Weight Gain (主题) OR Maternal Weight Gain (主题) OR Pregnancy Weight Gain (主题) OR Postpartum Weight Retention (主题) | 22206 |
| #2 | Search Pregnancy Outcome (主题) OR Pregnancy Outcome* (主题) OR birth outcome (主题) OR obstetric outcome (主题) | 228361 |
| #3 | Search Pregnancy Complications (主题) OR Pregnancy Complication (主题) OR Adverse Birth Outcome* (主题) OR complication* during pregnancy (主题) OR complications related to pregnancy (主题) OR disease* complicating pregnancy (主题) OR gestational complication* (主题) OR pregnancy neoplastic complications (主题) OR pregnancy-associated complications (主题) | 88054 |
| #4 | Add search(#2 OR #3) | 264823 |
| #5 | Add search(#4 AND #1) | 8123 |

**Table S5.** A systematic review was conducted on the 17 included studies using the modified Newcastle-Ottawa Scale.

| **Modiﬁed Newcastle–Ottawa scale** | | | | | | | | | |
| --- | --- | --- | --- | --- | --- | --- | --- | --- | --- |
| **Study** | **Representativeness of the exposed cohort** | **Selection of the nonexposed cohort** | **Ascertainment of exposure** | **Demonstration that outcome Of interest was not present at start of study** | **Comparability of cohorts on the basis of the design or analysis** | **Assessment of outcome** | **Was follow-up long enough** | **Adequacy of follow up of cohorts** | **Quality** |
| Monteiro, S. S. (18) | ⭐️ | ⭐️ | ⭐️ |  | ⭐⭐️️ | ⭐️ |  |  | 6 |
| Rosinha, P. M. O. (19) | ⭐️ | ⭐️ | ⭐️ |  | ⭐ | ⭐️ | ⭐️ | ⭐️ | 7 |
| Ukah, U. V. (20) | ⭐️ | ⭐️ |  |  | ⭐⭐️️ | ⭐️ | ⭐️ | ⭐️ | 7 |
| Yazan Arslan, A. (21) | ⭐️ | ⭐️ | ⭐ | ⭐️ | ⭐⭐️️ | ⭐️ | ⭐️ | ⭐️ | 9 |
| Langford, A. (22) | ⭐️ | ⭐️ | ⭐️ | ⭐️ | ⭐️ | ⭐️ | ⭐️ | ⭐️ | 8 |
| Liu, L. (23) | ⭐️ | ⭐️ | ⭐️ |  | ⭐️ | ⭐️ |  | ⭐️ | 6 |
| Gao, X. (24) | ⭐️ | ⭐️ | ⭐️ | ⭐️ | ⭐️ | ⭐️ | ⭐️ | ⭐️ | 8 |
| Shi, P. (25) | ⭐️ | ⭐️ | ⭐️ |  | ⭐ | ⭐️ | ⭐️ | ⭐️ | 7 |
| Wang, N. (26) | ⭐️ | ⭐️ | ⭐️ |  | ⭐ | ⭐️ | ⭐️ |  | 6 |
| Shao, Y. (27) | ⭐️ | ⭐️ | ⭐️ | ⭐️ | ⭐ | ⭐️ |  | ⭐️ | 7 |
| Vivatkusol, Y. (28) | ⭐️ | ⭐️ | ⭐️ |  | ⭐ | ⭐️ |  | ⭐️ | 6 |
| Zhang, L. (29) | ⭐️ | ⭐️ | ⭐️ | ⭐️ | ⭐ | ⭐️ | ⭐️ |  | 7 |
| Zhang, S. (30) | ⭐️ | ⭐️ | ⭐️ | ⭐️ | ⭐ | ⭐️ | ⭐️ |  | 7 |
| Omani-Samani, R. (31) | ⭐️ | ⭐️ | ⭐️ | ⭐️ | ⭐⭐️️ | ⭐️ | ⭐️ |  | 8 |
| Sámano, R. (32) | ⭐️ | ⭐️ | ⭐️ |  | ⭐️ | ⭐️ | ⭐️ |  | 6 |
| Almasi-Hashiani, A. (33) | ⭐️ | ⭐️ | ⭐️ |  | ⭐⭐️️ | ⭐️ | ⭐️ |  | 7 |
| Yin, B. B. (34) | ⭐️ | ⭐️ | ⭐️ | ⭐️ | ⭐ | ⭐️ |  | ⭐️ | 7 |
